# From the Editor’s Desk

**Published:** 2016

**Authors:** Patrick Commerford

**Affiliations:** Editor-in- Chief

It is interesting to one trained in the era that preceded the availability of new and sophisticated imaging modalities to see just how well the ‘old’ and often currently poorly regarded imaging techniques still serve. Ganie and colleagues (page 31) record how in seven of eight patients with vascular rings, the chest radiograph was abnormal, and they propose a diagnostic imaging algorithm for such patients, which includes chest radiographs. My own observations suggest that the chest radiograph is often neglected and may not be performed in current practice when patients are evaluated with symptoms or signs of cardiovascular disease, and are referred for echocardiography before a chest radiograph is performed. Echocardiography may fail to detect extra-cardiac abnormalities and mis-assess the severity of intracardiac abnormalities unless clinically directed. The authors are to be congratulated on pointing out how simple investigations, performed at low cost, may be valuable in evaluating patients with complex cardiovascular abnormalities.

In exploring the genetic contribution to hypertension, Sun and colleagues (page 21) have identified genes that were risk factors for nocturnal hypertension in this Chinese Han population, and their combined effects played an important role in nocturnal hypertension. However, as the authors acknowledge, even if a gene were considered associated with hypertension in certain populations, to expand the conclusion to all human populations is unwise at this stage. In diseases such as hypertension, obesity or diabetes, not only genetic factors but many other factors, such as environment or geographic location, could be important. All these factors could have different effects on obesity or diabetes and they could impact on each other. Therefore whether or how single genes may be associated with nocturnal hypertension is complicated. Long-term population-based studies are needed to clarify the relationship.

Arodiwe and co-authors (page 26) point out the paucity of information on the frequency of cardiac involvement in children with HIV/AIDS in sub-Saharan Africa, and the importance of such information given the extent of the epidemic. They conducted a descriptive cross-sectional echocardiographic study of HIV-infected children and uninfected controls at a Nigerian teaching hospital and showed left ventricular systolic dysfunction in 27% of HIV-infected children and 81% of those with AIDS. The systolic dysfunction was asymptomatic. This is important information and hopefully there will be further information forthcoming from the authors regarding follow up and prognosis of this particular group of patients, as well as the effects of anti-retroviral therapy. The impact of usual cardio-active medication for systolic dysfunction, such as angiotensin converting enzyme inhibitors and beta-blockers, should also be explored in this population.

The clinical, laboratory and ECG profiles of 62 children with sickle cell anaemia (SCA) attending a paediatric haematology clinic in Nigeria and 40 age- and gender-matched haemoglobin AA controls were compared (page 16). Adegoke and colleagues found that ECG abnormalities were common in children with SCA, and in their discussion, speculate on the mechanisms and significance of the ECG abnormalities.

